# Oropharyngeal Dysphagia as a Metabolic Emergency: A Comprehensive Review on Nutritional Barriers, Sarcopenia, and Management Strategies

**DOI:** 10.3390/nu18121940

**Published:** 2026-06-16

**Authors:** Sebastiano Mercadante

**Affiliations:** Main Regional of Supportive/Palliative Care, La Maddalena Cancer Center, Via San Lorenzo 312, 90146 Palermo, Italy; mercadante.sebastiano@lamaddalenanet.it or terapiadeldolore@lamaddalenanet.it or 03sebelle@gmail.com

**Keywords:** oropharyngeal dysphagia, malnutrition, sarcopenia, nutritional management, IDDSI, texture-modified diet, enteral nutrition

## Abstract

Oropharyngeal dysphagia (OD) is traditionally managed as a mechanical swallowing impairment. This narrative review proposes a conceptual model that reframes chronic, severe OD as a high-risk clinical condition driving systemic malnutrition and progressive nutritional deterioration. We examine the epidemiological burden of OD-associated malnutrition across geriatric, neurological, and oncological populations, exploring how diagnostic heterogeneity influences reported prevalence ranges. The pathophysiological narrative synthesizes hypotheses regarding the potential disruption of the cephalic phase of digestion, the rheological limitations of texture-modified diets (TMDs), and the theoretical bioenergetic cost of impaired swallowing. Central to this review is the hypothetical sarcopenia–dysphagia vicious cycle, evaluating how molecular pathways—such as systemic inflammation, ubiquitin–proteasome-mediated proteolysis, and suppression of muscle protein synthesis—are inferred from broader cachexia models to affect oropharyngeal function. We discuss structured nutritional management strategies, including micro-volume fortification, application of the IDDSI framework with xanthan gum-based thickeners, and monitoring via GLIM criteria, bioelectrical impedance analysis, and routine laboratory parameters. Finally, we analyze the ethical challenges of transitioning to enteral nutrition and outline the translational limitations of emerging fields like 3D food printing. This model aims to encourage clinical focus on comprehensive nutritional restoration alongside airway safety.

## 1. Introduction

Traditionally, clinical literature has classified oropharyngeal dysphagia (OD) as a functional sequela of aging (presbyphagia) or as a symptomatic byproduct of neurological and structural disorders, such as stroke, Parkinson’s disease, or head and neck cancer. However, expanding clinical evidence suggests that OD may be more accurately defined as a primary driver of malnutrition (MN) and a significant metabolic obstacle to systemic homeostasis [1]. Emerging paradigms suggest classifying chronic OD as a major geriatric syndrome, acknowledging that its clinical impact extends beyond the mechanics of the bolus to the progressive depletion of the host’s nutritional reserves [2].

OD often acts as an insidious determinant of nutritional status. Although the acute clinical focus in dysphagia management typically targets the prevention of aspiration pneumonia—a critical concern given its high mortality rate—the chronic, cumulative deficit in macro- and micronutrients is frequently overlooked [3]. This long-term nutritional erosion is hypothesized to precipitate frailty, secondary sarcopenia, and increased vulnerability to infections. The physical inability to ingest food efficiently can establish a chronic undernutrition status, which compromises immune function and theoretically amplifies the risk of respiratory complications [4].

In this review, we propose a conceptual model reframing severe, unmanaged OD as a state of “metabolic emergency”. Rather than implying an acute, universally accepted metabolic diagnosis, this term is used as a conceptual framework to denote a high-risk clinical condition characterized by rapid or progressive nutritional deterioration. The clinical urgency of this condition depends heavily on the patient’s acute or chronic status. In acute settings, such as the early phase of an ischemic stroke, the sudden onset of neurological dysphagia overlaps with injury-induced hypermetabolism, constituting an acute catabolic stress that requires rapid nutritional intervention to protect lean mass. Conversely, in chronic neurodegenerative disorders or community-dwelling older adults, the condition manifests as a progressive multi-organ decline driven by a chronic volumetric and calorie-protein gap [5]. Identifying OD is therefore not only a task for functional rehabilitation but also a key step in preventing systemic nutritional depletion. Presenting this concept as a proposed model may help clinicians balance the immediate goals of swallowing safety with the long-term objective of comprehensive metabolic restoration.

### Literature Search Strategy and Selection Criteria

To ensure a structured and balanced overview, a literature search was conducted across PubMed/MEDLINE, Scopus, and the Cochrane Library for articles published up to June 2026. The search utilized combinations of Medical Subject Headings (MeSH) terms and keywords, including “oropharyngeal dysphagia”, “malnutrition”, “sarcopenia”, “metabolic expenditure”, “texture-modified diets”, and “enteral nutrition”. Preference was given to international consensus guidelines (e.g., European Society for Clinical Nutrition and Metabolism [ESPEN], European Working Group on Sarcopenia in Older People [EWGSOP2]), systematic reviews, meta-analyses, and randomized controlled trials. Representative narrative reviews, retrospective analyses, and expert opinion documents were integrated when high-tier clinical trial data were limited.

## 2. Epidemiological Links: Malnutrition and Risk

The intersection of OD and MN is highly variable across clinical settings and underlying pathologies. However, observational data consistently indicate that the presence of OD escalates the risk of developing MN by a factor of three to five compared to non-dysphagic peers [5]. This correlation is hypothesized to be bidirectional and synergistic: chronic undernutrition weakens the oropharyngeal musculature, which directly exacerbates the severity of the dysphagia, creating a functional and nutritional decline. This epidemiological burden is further compounded by secondary sarcopenia, where systemic inflammation and reduced protein intake lead to a rapid depletion of muscle reserves, theoretically impacting the safety and efficiency of the swallow reflex.

A major challenge in evaluating the epidemiological literature is the wide variance in reported prevalence ranges (e.g., Parkinson’s disease: 35–80%; stroke: 50–78%) (Table 1). This broad variability is primarily driven by significant diagnostic heterogeneity across studies. Lower prevalence figures are typically associated with subjective bedside screening questionnaires or self-reported symptom scales, which frequently underdiagnose silent aspiration or mild functional impairments. Conversely, substantially higher prevalence rates are reported when studies utilize objective, instrumental gold standards, such as Videofluoroscopic Swallow Study (VFSS) or Fiberoptic Endoscopic Evaluation of Swallowing (FEES). Population differences, varying stages of disease severity, and the specific clinical setting (acute care versus long-term institutionalization) further contribute to this statistical variance.

### 2.1. The Geriatric Population and “Presbyphagia”

In the community-dwelling elderly, the prevalence of OD is estimated to be between 15% and 30% [1]. However, in nursing homes and long-term care facilities, this figure surges to over 60% [6,7]. Observational studies indicate that malnutrition ranges between 80% and 90% among institutionalized elderly patients with documented dysphagia [5,8]. A primary nutritional driver in this setting is the “dilution effect” often associated with texture-modified diets (TMDs) [8,15]. To achieve a safe bolus consistency, dense or solid foods are frequently diluted with liquids or mechanically modified into purees, which can increase the total volume of the meal while substantially decreasing its caloric and protein density. This volume expansion can cause early satiety via gastric distension before the patient’s actual macro- and micronutrient requirements are met. This phenomenon is particularly challenging in older adults who already face age-related reductions in appetite (anorexia of aging), making the transition to modified textures a potential gateway to sarcopenic dysphagia [5].

### 2.2. Stroke and Acute Neurological Injury: The Sarcopenia–Dysphagia Nexus

In the acute phase of stroke, OD affects approximately 50% to 78% of patients, and the nutritional fallout is immediate and profound [9]. Patients with post-stroke OD are disproportionately affected by dehydration and rapid muscle wasting, as the acute inflammatory response to the brain injury increases metabolic demands while the physical barrier of dysphagia prevents adequate intake.

The relationship between stroke and muscle loss is increasingly recognized as “stroke-related sarcopenia”, a condition driven by systemic inflammation and sympathetic overactivation. In this context, the stroke induces a neurogenic weakness of the swallowing muscles (primary damage), while the subsequent MN and systemic catabolism trigger a rapid loss of skeletal muscle mass (secondary sarcopenia). It is mechanistically plausible that this secondary loss affects the suprahyoid and infrahyoid musculature essential for laryngeal elevation. However, it must be noted that primary literature, such as reference [10], evaluates systemic post-stroke sarcopenia; selective, accelerated atrophy of the swallowing muscles remains a speculative inference that requires direct, localized validation in human clinical trials. The systemic release of pro-inflammatory cytokines (tumor necrosis factor-alpha [TNF-α], interleukin-6 [IL-6]) promotes protein breakdown, meaning that if a patient is kept in prolonged nil per os (NPO) status without aggressive nutritional support, the resulting sarcopenia can transform a transient deficit into a chronic disability [10].

### 2.3. Neurodegenerative Diseases: Parkinson’s and Dementia

In Parkinson’s disease (PD), OD eventually affects up to 80% of patients. The link to malnutrition is exacerbated by “off-periods” and tremors that significantly increase resting energy expenditure. The act of eating becomes an exhausting physical task; the coordination required during periods of bradykinesia leads to reduced caloric intake and “muscle fatigue dysphagia” [11,12]. In dementia, OD is frequently accompanied by feeding difficulties and apraxia of swallowing. As the disease progresses, the patient enters a state of “neurogenic sarcopenia”, where the lack of central motor drive leads to disuse atrophy of the pharyngeal muscles. This is compounded by behavioral symptoms (pacing, agitation) that increase energy needs while the patient is physically unable to safely consume dense nutrients [13].

### 2.4. Head and Neck Cancer (HNC) and Esophageal Cancer

OD in HNC is often iatrogenic, resulting from surgical resection or radiotherapy-induced fibrosis [14]. These patients face cancer cachexia accelerated by mechanical obstruction. Studies show that up to 75% of HNC patients experience significant weight loss (>10% of body weight) during treatment, driven by OD, mucositis, and xerostomia [16,17].

While esophageal cancer primarily presents as mechanical esophageal dysphagia rather than functional oropharyngeal dysphagia, it shares identical catabolic consequences. Malignant luminal obstruction typically follows a progressive pattern, reducing intake from solids to liquids as the diameter drops below 10–12 mm [18,19]. In advanced stages, complete aphagia can occur, which is strongly associated with an accelerated cachectic decline and shortened survival [20]. Restoring luminal patency via self-expanding metal stents (SEMS) serves as a paradigm for rapid nutritional rehabilitation, transitioning patients to modified soft diets within 24–48 h [20,21].

### 2.5. Bidirectional Link: Sarcopenia and Dysphagia

Systemic loss of skeletal muscle mass affecting the swallowing muscles is termed sarcopenic dysphagia [22]. Systemic sarcopenia leads to oropharyngeal weakness, which compromises energy and protein intake, further exacerbating systemic muscle wasting [23]. Chronic undernutrition prevents the activation of the mTORC1 pathway, the primary molecular driver of muscle protein synthesis [24].

## 3. Pathophysiology of Nutritional Intake in OD

The nutritional deficit in OD is not merely a quantitative problem of insufficient food bolus transit; it represents a comprehensive disruption of the digestive–absorptive axis. This disruption begins long before the bolus reaches the stomach, affecting the enzymatic, hormonal, and mechanical efficiency of the entire gastrointestinal tract [1]. In the context of a proposed “metabolic emergency” model, severe OD may be viewed as a state of digestive dys-synchrony, where the normal anticipatory signals of the gut–brain axis are potentially mismatched with actual nutrient delivery.

### 3.1. The Compromised Cephalic Phase of Digestion (Hypothetical Mechanisms)

The digestion process is classically divided into three phases: cephalic, gastric, and intestinal. The cephalic phase is responsible for approximately 20% to 30% of total gastric acid secretion in response to visual, olfactory, and gustatory stimuli [25]. In patients with severe, chronic OD, this phase is hypothesized to be severely blunted or entirely bypassed.

Texture-modified diets (TMDs), particularly pureed foods (IDDSI Level 4), often suffer from a loss of visual identity and olfactory appeal. This “sensory monotony” has been proposed to cause a reduction in initial salivary flow and a diminished secretion of gastric acid and pepsinogen [25]. Furthermore, the lack of mastication—which normally stimulates the periodontal–parotid reflex—results in lower concentrations of salivary epidermal growth factor (EGF), potentially delaying the repair of the esophageal and gastric mucosa in fragile patients.

It has been hypothesized in broader nutritional literature that sensory restriction and lack of oral signaling could affect the secretion of appetite-regulating hormones, such as flattening physiological ghrelin and leptin oscillations, leading to the “anorexia of dysphagia” [25], though direct clinical documentation in dysphagic populations remains limited. Similarly, a blunting of the cephalic phase insulin release (CPIR) has been inferred, which could theoretically impair early glucose tolerance [26]. However, it must be emphasized that while these physiological responses are well-documented in healthy subjects exposed to sensory restriction models, direct human evidence tracking ghrelin, leptin, and CPIR alterations specifically within dysphagic cohorts remains highly limited; these mechanisms should currently be treated as unproven theoretical extrapolations.

### 3.2. Bolus Rheology and the Starch–Amylase Interaction: Inferences

The science of rheology is fundamental to understanding why dysphagia management can fail nutritionally. The modification of food consistency introduces significant biochemical barriers that alter nutrient bioavailability [27]. Starch-based thickening agents are highly susceptible to salivary alpha-amylase. In vitro and general physiological studies demonstrate that even trace amounts of saliva can rapidly degrade the viscosity of a starch-thickened liquid within seconds [28]. This can create a rapid viscosity drop mid-swallow, potentially increasing aspiration risk.

Based on general carbohydrate rheology, it has been inferred that this rapid enzymatic hydrolysis could alter the glycemic index of the meal, causing rapid glucose spikes and subsequent reactive hypoglycemia, which might further suppress appetite [29]. However, there is currently a lack of direct human trials mapping glycemic curves or reactive hypoglycemia explicitly in patients with OD consuming starch thickeners; this pathway remains a logical inference rather than an established clinical fact. Conversely, while xanthan gum-based thickeners resist amylase degradation, the high viscosity of IDDSI Level 3 or 4 liquids can slow down gastric emptying in general models. This prolonged gastric residence time may maintain a state of mechanoreceptor distension that signals fullness to the hypothalamus, potentially contributing to early satiety and a subsequent reduction in the intake of nutrient-dense solid foods [29].

### 3.3. The Mechanical Cost of Eating: Fatigue and Catabolism

The energetic cost of the meal itself is an important factor to consider in OD-related malnutrition. In a healthy state, the energy expended during eating is negligible; in severe OD, it can become a measurable mechanical drain [30]. For a patient with pharyngeal weakness, the mechanical effort required to clear a bolus requires an exaggerated recruitment of the suprahyoid musculature and accessory neck muscles.

Clinical observations indicate that when a mealtime exceeds 30 to 45 min due to these impairments, the total volume of food consumed drops precipitously because of physical exhaustion of the deglutition muscles. Mathematical modeling and general nutritional estimations suggest this “fatigue-induced calorie gap” can lead to a cumulative daily deficit of 300–500 kcal in highly compromised patients [31]. In individuals already experiencing catabolic stress, this prolonged negative energy balance accelerates systemic muscle proteolysis, further weakening the swallowing apparatus and contributing to a progressive functional decline.

## 4. Musculoskeletal Consequences: The Sarcopenia–Malnutrition Feedback Loop

OD is hypothesized to inflict a coordinated stress on the musculoskeletal system, creating a feedback loop between systemic frailty and localized functional failure [11]. Unlike simple starvation, where fat stores are primarily mobilized, the nutritional crisis in severe OD is frequently accompanied by an inflammatory or neurological stressor that prioritizes the breakdown of lean body mass. This metabolic priority can shift the body into a pro-catabolic state where the muscles of the head and neck are sacrificed to maintain systemic amino acid pools.

### 4.1. The Sarcopenia–Dysphagia Vicious Cycle

Sarcopenia is characterized by the progressive loss of skeletal muscle mass, strength, and physical performance [32]. When this systemic decline affects the specific muscles of deglutition—specifically the masseters, the suprahyoid complex, and the pharyngeal constrictors—it is termed sarcopenic dysphagia [22].

The relationship is bidirectional. Systemic sarcopenia leads to oropharyngeal weakness, which increases the time and effort required for swallowing; this weakness compromises total energy and protein intake, which in turn exacerbates malnutrition and further systemic muscle wasting [23]. In the elderly, this is often compounded by “disuse atrophy” of the swallowing muscles due to prolonged reliance on pureed diets or enteral nutrition.

Chronic undernutrition, combined with age-related anabolic resistance, prevents the repair and hypertrophy of muscle tissue. The lack of essential amino acids, particularly Leucine, prevents the activation of the mTORC1 pathway, the primary molecular driver of muscle protein synthesis (MPS) [33]. Without sufficient mTORC1 signaling, the body cannot counteract the daily rate of muscle protein breakdown, leading to a net negative protein balance that specifically erodes the functional reserve of the upper aerodigestive tract [24].

### 4.2. Molecular Mechanisms: Proteolysis and Inflammation (Extrapolated Concepts)

The transition from simple weight loss to a state of profound malnutrition in OD is mediated by complex molecular signaling pathways. It has been hypothesized that in patients with chronic OD, recurrent silent microaspiration could trigger a low-grade localized or systemic inflammatory state, increasing circulating pro-inflammatory cytokines such as IL-6 and TNF-alpha [11]. In general muscle biology, these inflammatory markers act as catalysts for muscle degradation by activating the ubiquitin–proteasome pathway. TNF-alpha, in particular, stimulates the expression of muscle-specific E3 ubiquitin ligases, such as muscle RING finger 1 (MuRF1) and atrogin-1, leading to the targeted degradation of myofibrillar proteins [34].

Ultrasonographic and electromyographic studies in general aging cohorts indicate that the geniohyoid muscle and the tongue base—which are rich in Type II (fast-twitch) fibers—are highly sensitive to systemic protein depletion and inflammatory signaling [35]. Because these fibers are responsible for the rapid, forceful movements required for laryngeal elevation and bolus propulsion, their loss explains why dysphagic patients frequently exhibit a delayed or weakened swallow reflex [36]. However, clinicians must note that a direct, unarguable causal link demonstrating that silent microaspiration directly activates the MuRF1/atrogin-1 pathway specifically within human swallowing muscles has not been conclusively established; this comprehensive pathway is a highly plausible mechanistic model inferred from broader sarcopenia and cachexia literature.

## 5. Nutritional Management Strategies

The clinical management of OD presents a fundamental therapeutic paradox: the rheological modifications required to ensure airway safety inherently act as barriers to nutritional adequacy. To resolve this, a multimodal nutritional strategy is required, shifting the focus from simple “thickening” to metabolic optimization [31,36].

### 5.1. The Science of Fortification: Nutrient Density over Volume

In the dysphagic population, especially the elderly or those with neurological frailty, gastric capacity is often limited, and mealtime fatigue prevents the consumption of large volumes. Therefore, a primary intervention strategy is high-density micro-volume fortification [31]. Lipids represent an efficient substrate for energy enrichment. Medium-chain triglycerides (MCTs) are absorbed directly into the portal circulation without the need for bile acid emulsification or pancreatic lipase. Incorporating MCTs into pureed meals (IDDSI Level 4) can increase caloric density by 45–90 kcal per 100 g without altering the flavor or viscosity [37].

To counteract sarcopenia, international consensus guidelines (e.g., ESPEN) recommend elevating protein intake to 1.2–1.5 g/kg/day in older adults [31]. Whey protein isolates (WPIs) are a preferred substrate due to their rapid digestion kinetics and high concentration of Leucine, which acts as a potent signaling molecule that activates the mTORC1 pathway for muscle protein synthesis [33].

### 5.2. Rheological Engineering: The IDDSI Framework

The IDDSI framework has revolutionized management by providing a standardized, global language for viscosity and texture [38]. Modified starches are highly vulnerable to salivary α-amylase, which degrades viscosity in seconds (amylase thin-out). In contrast, xanthan gum (XG) thickeners create a stable, non-Newtonian fluid resistant to amylase, ensuring bolus stability throughout the pharyngeal phase [28].

While water in XG matrices is bioavailable in the small intestine, the reduced “thirst-quenching” sensation and the altered mouthfeel of thickened liquids often lead to subclinical dehydration. This dehydration increases the risk of delirium, urinary tract infections, and acute kidney injury [39].

### 5.3. Oral Nutritional Supplements (ONS) and Medical Foods

When oral intake fails to meet 75% of requirements for more than 5–7 days, ONS must be introduced to close the calorie–protein gap [31]. These pre-thickened ONS (IDDSI Level 4) can provide up to 2.4 kcal/mL. This format mirrors a pudding or yogurt, making it more socially acceptable and easier to manage for patients with pharyngeal delay or reduced tongue pressure [40].

The success of nutritional management must be tracked using the Global Leadership Initiative on Malnutrition (GLIM) criteria. Moving beyond simple body weight, clinicians should utilize Bioelectrical Impedance Analysis (BIA). Monitoring parameters like phase angle (PhA) provides a direct window into cellular integrity and muscle quality [41].

In addition to phenotypic assessment, comprehensive nutritional monitoring in older hospitalized patients should incorporate objective clinical and laboratory parameters. Routine hematological, biochemical, and immunological indicators are crucial for early risk identification. Serum albumin and prealbumin tiers reflect visceral protein reserves, while a complete blood count evaluates hemoglobin status and absolute lymphocyte count, aiding the identification of systemic inflammation and immune vulnerability secondary to chronic undernutrition [42].

## 6. Clinical Application: Pathophysiology-Based Nutritional Vignettes

The following clinical scenarios are presented strictly as hypothetical, illustrative examples designed to demonstrate how clinicians might interpret and apply broader nutritional literature to individual patient profiles, rather than validated clinical algorithms or guideline-mandated protocols.

### 6.1. Case Scenario A: The Post-Stroke Acute Patient

A 72-year-old male with severe OD following an ischemic stroke faces a state of acute neuroinflammation and hypermetabolism [9,10]. In this phase, the patient is at the peak of the catabolic surge.

In this scenario, implementing an individualized, expert-supported “bridge strategy”—combining parenteral hydration with early fortified oral nutrition—serves as an illustrative approach to safely achieve guideline-driven targets. This involves utilizing IDDSI Level 4 meals fortified with WPIs to aggressively target a protein intake of 1.5 g/kg/day within the first 72 h, a strategy theoretically designed to mitigate rapid disuse atrophy of vital swallowing musculature [43].

### 6.2. Case Scenario B: Advanced Parkinson’s Disease

An 80-year-old female with Parkinson’s disease (PD) experiences tremors and dyskinesia that can double her basal metabolic rate [44]. The strategy shifts to high-calorie, small-volume ONS (2.4 kcal/mL) delivered strategically during “on-periods” when coordination is optimized. The integration of MCT oils to provide calorie enrichment offers a clean energy source that theoretically does not interfere with the intestinal absorption of levodopa [45].

### 6.3. Case Scenario C: Head and Neck Cancer (HNC)

A 55-year-old male undergoing radiotherapy for base-of-tongue carcinoma experiences severe iatrogenic OD, exacerbated by radiation-induced mucositis and xerostomia [14,16].

This complex profile can be managed via an evidence-supported, patient-specific “triple-track” approach. Proactive placement of a percutaneous endoscopic gastrostomy (PEG) tube is performed to secure baseline caloric and fluid needs [46,47,48,49,50,51,52,53,54]. Simultaneously, to prevent disuse muscular atrophy, the patient continues supportive “sensory” oral intake with IDDSI Level 4 purees enriched with Omega-3 fatty acids, which play an expert-opinion-based role in potentially mitigating mucosal inflammation [55].

## 7. Ethical Management and the Transition to Artificial Nutrition

The transition from oral to artificial nutrition and hydration (ANH) represents one of the most complex decision-making processes in the clinical management of OD [47].

### 7.1. Enteral Nutrition (EN): Timing and Rationale

When oral intake remains below 75% of requirements for more than 7–14 days, EN must be considered [48]. A nasogastric tube (NGT) is preferred for short-term management (<4 weeks). PEG is indicated for long-term support [49]. EN should be viewed as a “metabolic bridge” that maintains the muscular strength required for active swallowing rehabilitation [50].

### 7.2. The Ethical Dilemma in Advanced Dementia

In patients with advanced dementia, the use of ANH is a subject of significant ethical debate. Clinical evidence suggests that PEG feeding in advanced dementia does not significantly prolong life or improve functional status [51]. For this population, the nutritional perspective shifts toward careful hand feeding (CHF), prioritizing hedonic and sensory value [52].

### 7.3. Refeeding Syndrome and Metabolic Monitoring

Any transition to ANH in a severely malnourished dysphagic patient carries the risk of refeeding syndrome [53]. The rapid introduction of carbohydrates triggers a massive insulin release, causing severe hypophosphatemia, hypomagnesemia, and hypokalemia. Nutritional protocols must emphasize a “start slow, go slow” approach, beginning at 10–15 kcal/kg/day with daily electrolyte monitoring [53,54].

## 8. Future Directions: The Microbiome and Digital Gastronomy

The future of OD management lies in the integration of personalized medicine, molecular biology, and advanced food engineering [55].

### 8.1. The Gut–Lung–Oropharynx Axis and the Microbiome: Limitations

Chronic OD often involves oral dysbiosis, where the pulmonary inflammatory response may be modulated by a pathogenic bacterial load from microaspiration [56]. Furthermore, reduced fiber intake leads to gut dysbiosis and a reduction in short-chain fatty acids (SCFAs), which is hypothesized to exacerbate anabolic resistance in the swallowing muscles [57]. However, research in this field faces substantial translational challenges, and therapeutic interventions like targeted probiotics or SCFA modifications remain in early laboratory stages.

### 8.2. 3D Food Printing (3DFP): Translational Challenges

3DFP allows for the reconstruction of pureed foods into complex, recognizable shapes that mimic original textures [58]. It has been conceptually proposed that restoring the visual architecture of a meal could help re-trigger the cephalic phase of digestion, potentially improving the early enzymatic response to food intake [25,59]. However, current research primarily demonstrates conceptual and technological possibilities. Evidence showing improved clinical outcomes or direct activation of the cephalic phase in dysphagic patients is currently limited. Furthermore, significant translational challenges—including high equipment costs, institutional feasibility constraints, strict rheological standardization needs, and microbiological safety concerns—must be resolved before 3DFP can be considered a viable tool in routine clinical practice.

## 9. Conclusions: Reclassifying OD as a Systemic Metabolic Challenge

In conclusion, this analysis underscores that oropharyngeal dysphagia represents a significant nutritional and metabolic challenge. Presenting the concept as a proposed model emphasizes that OD is an insidious determinant of systemic malnutrition [4,15]. The inferred sarcopenia–dysphagia cycle highlights the potential utility of aggressive protein and lipid optimization [32,36]. Clinical management must remain multimodal, integrating IDDSI standards, stable xanthan gum thickeners, and nutrient fortification [28,38], while tracking outcomes through phenotypic and objective laboratory markers [7,42,43]. Shifting the clinical paradigm from an exclusive focus on “swallowing safety” to a balanced model incorporating “metabolic restoration” is important to optimize clinical outcomes and minimize functional dependency.

## Figures and Tables

**Table 1:** Prevalence of oropharyngeal dysphagia (OD) and malnutrition (MN) across different clinical settings. Note: The broad prevalence ranges reported reflect significant heterogeneity in the literature regarding diagnostic methodologies (e.g., subjective screening tools versus instrumental videoendoscopy), clinical settings, and underlying disease severities.

Clinical Population	Prevalence of OD (%)	Prevalence of MN Patients (%)	Primary Nutritional Risk Factor
Community Elderly	15–30% [6]	35–45% [7]	Phagophobia & Isolation
Nursing Homes	60–70% [6]	80–90% [8]	Low-Density TMD
Acute Stroke	50–78% [9]	40–55% [10]	Hypermetabolism
Parkinson’s	35–80% [11]	15–30% [12]	Energy Expenditure
Head & Neck Cancer	50–90% [13]	65–75% [14]	Mucositis & Fibrosis

## Data Availability

No new data were created or analyzed in this study. Data sharing is not applicable to this article.
